# Effect of L-DOPA/Benserazide on Propagation of Pathological α-Synuclein

**DOI:** 10.3389/fnins.2019.00595

**Published:** 2019-06-14

**Authors:** Aki Shimozawa, Yuuki Fujita, Hiromi Kondo, Yu Takimoto, Makoto Terada, Masanao Sanagi, Shin-ichi Hisanaga, Masato Hasegawa

**Affiliations:** ^1^Department of Dementia and Higher Brain Function, Tokyo Metropolitan Institute of Medical Science, Tokyo, Japan; ^2^Department of Biological Science, Tokyo Metropolitan University, Tokyo, Japan; ^3^Discovery Service, Charles River Laboratories Japan, Inc., Ibaraki, Japan

**Keywords:** α-synuclein, Parkinson’s disease, propagation, L-DOPA, benserazide

## Abstract

Parkinson’s disease (PD) and related disorders are characterized by filamentous or fibrous structures consisting of abnormal α-synuclein in the brains of patients, and the distributions and spread of these pathologies are closely correlated with disease progression. L-DOPA (a dopamine precursor) is the most effective therapy for PD, but it remains unclear whether the drug has any effect on the formation and propagation of pathogenic abnormal α-synuclein *in vivo*. Here, we tested whether or not L-DOPA influences the prion-like spread of α-synuclein pathologies in a wild-type (WT) mouse model of α-synuclein propagation. To quantitative the pathological α-synuclein in mice, we prepared brain sections stained with an anti-phosphoSer129 (PS129) antibody after pretreatments with autoclaving and formic acid, and carefully analyzed positive aggregates on multiple sections covering the areas of interest using a microscope. Notably, a significant reduction in the accumulation of phosphorylated α-synuclein was detected in substantia nigra of L-DOPA/benserazide (a dopamine decarboxylase inhibitor)-treated mice, compared with control mice. These results suggest that L-DOPA may slow the progression of PD *in vivo* by suppressing the aggregation of α-synuclein in dopaminergic neurons and the cell-to-cell propagation of abnormal α-synuclein. This is the first report describing the suppressing effect of L-DOPA/benserazide on the propagation of pathological α-synuclein. The experimental protocols and detection methods in this study are expected to be useful for evaluation of drug candidates or new therapies targeting the propagation of α-synuclein.

## Introduction

Parkinson’s disease (PD) is the second most common neurodegenerative disease after Alzheimer’s disease, and is characterized by the appearance of Lewy bodies (LBs) and Lewy neurites (LNs). Dementia with Lewy bodies (DLB) is also a progressive neurodegenerative disease, in which LBs and LNs appear in the cortex (Goedert, 2001; Hasegawa et al., 2016). Mutations in the α-synuclein gene *SNCA* were found to be associated with these diseases, and subsequent immunostaining studies with antibodies demonstrated that α-synuclein is the major component of LBs and LNs (Spillantini et al., 1997, 1998b; Baba et al., 1998). It is also the major component of glial cytoplasmic inclusions (GCIs) in multiple system atrophy (MSA) (Spillantini et al., 1998a; Wakabayashi et al., 1998). α-Synuclein is a small protein of 140 amino acids, which is localized in presynaptic termini, and is involved in the maintenance of synapses and in synaptic plasticity. However, in patients with these α-synucleinopathies (PD, DLB, or MSA), abnormal filamentous α-synuclein deposits with cross-β structure appear in the brain. This α-synuclein is phosphorylated at Ser129 and partially ubiquitinated (Fujiwara et al., 2002; Hasegawa et al., 2002). Thus, phosphorylated α-synuclein represents the pathological or abnormal form of α-synuclein, and it can be detected with the PS129 antibody. Moreover, the spread of pathological α-synuclein is closely correlated with disease progression; indeed, the distribution pattern and spread of the pathologies are used for staging of sporadic PD (Braak et al., 2003; Saito et al., 2003). To date, six missense mutations in the *SNCA* gene and the occurrence of gene multiplication have been identified in familial forms of PD and DLB (Polymeropoulos et al., 1997; Kruger et al., 1998; Singleton et al., 2003; Chartier-Harlin et al., 2004; Ibanez et al., 2004; Zarranz et al., 2004; Appel-Cresswell et al., 2013; Lesage et al., 2013). These pathogenic mutations have various effects on fibril formation *in vitro*, either accelerating fibril formation (Conway et al., 1998; Choi et al., 2004; Ono et al., 2011; Ghosh et al., 2013) or resulting in the formation of fibrils that are more fragile and easier to propagate than wild-type (WT) fibrils (Yonetani et al., 2009). These results suggest that intracellular amyloid-like α-synuclein fibrils can cause PD and DLB, and the spread of α-synuclein pathology in the brain is considered to be the underlying mechanism of progression of these diseases. Indeed, recent experimental studies have demonstrated that intracerebral injection of synthetic α-synuclein fibrils and/or insoluble α-synuclein from diseased brain converts normal α-synuclein into abnormal form, and the abnormal α-synuclein propagates throughout the brain in a prion-like manner in α-synuclein transgenic mouse (Luk et al., 2012b; Watts et al., 2013), WT mouse (Luk et al., 2012a; Masuda-Suzukake et al., 2013, 2014; Tarutani et al., 2016) and WT marmoset (Shimozawa et al., 2017).

L-DOPA has been the gold-standard treatment for PD, since the 1960s. It ameliorates the three major motor signs (resting tremor, akinesia, and rigidity) of PD, although improvement of tremor by L-DOPA is mild compared to other anti-Parkinsonian drugs. Administration of a sufficient amount of L-DOPA, which serves as a precursor of dopamine, can maintain motor functions for a long time, leading to improved quality of life (QOL) and prolongation of survival time of the patients. Despite the effect of levodopa in reducing the symptoms of Parkinson’s disease, concern had been expressed that its use might hasten neurodegeneration. However, a multicenter, placebo-controlled, randomized, dose-ranging, double-blind clinical trial, called the Earlier versus Later Levodopa Therapy in Parkinson’s Disease (ELLDOPA) study, demonstrated that the total scores increased more in the placebo group than in all the groups receiving levodopa. A strong dose–response benefit was detected during the period, and the effect persisted through week 40 in the group receiving the highest dose of levodopa (600 mg daily), indicating that treatment with the higher dose of L-DOPA is beneficial (Fahn et al., 2004). Thus, this study and numerous other studies have established the effects of L-DOPA on the clinical symptoms of PD and other diseases, as well as its side effects. However, it remains to be clarified whether the drug has any effect on the *in vivo* formation and propagation of pathogenic abnormal α-synuclein, the key molecule in the pathogenesis and progression of the disease. L-DOPA is normally administered with a dopamine decarboxylase inhibitor, such as benserazide or carbidopa, to prevent conversion of L-DOPA to dopamine in the bloodstream, because dopamine cannot cross the blood-brain barrier. In this study, benserazide was used to inhibit the decarboxylation to dopamine and minimize the occurrence of extracerebral side effects of dopamine.

The aim of this study was to investigate whether L-DOPA/benserazide influences the *in vivo* aggregation of α-synuclein and whether it can slow the propagation of pathological α-synuclein. We present evidence that oral administration of L-DOPA in combination with benserazide reduced the accumulation of phosphorylated α-synuclein in substantia nigra in a WT mouse model injected with preformed synthetic α-synuclein fibrils. The results suggest that L-DOPA may indeed work, at least in part, by inhibiting the propagation of pathogenic protein *in vivo*.

## Materials and Methods

### Preparation of Recombinant α-Synuclein and Fibrils

Recombinant mouse α-synuclein and the fibrils were prepared as described previously, with minor modifications (Masuda-Suzukake et al., 2014; Tarutani et al., 2016). Briefly, purified mouse α-synuclein (7∼10 mg/ml) was incubated at 37°C in a shaking incubator at 200 rpm in 30 mM Tris–HCl, pH 7.5, containing 0.1% NaN_3_, for 72 h. α-Synuclein fibrils were pelleted by ultracentrifugation at 113,000 × *g* for 20 min, washed with sterile saline, resuspended in sterile saline (pH 7.5), and sonicated for 3 min in a Cup Horn type sonicator (Branson, SFX250) at 25°C. Protein concentrations were determined by HPLC.

### Intracerebral Injection of α-Synuclein Fibrils and Oral Administration of Drugs

C57BL/6N WT male mice were used for this experiment. Under anesthesia with ketamine hydrochloride (71.5 mg/kg, i.p.) and xylazine (14.5 mg/kg, i.p.), the animals were injected with 5 μL of 2 mg/mL mouse α-synuclein fibrils into the striatum (bregma +0.5 mm, 2 mm, and 3.5 mm) in the right hemisphere. Appropriate doses of viccillin and meloxicam were administered perioperatively to reduce the incidence of postoperative wound infections and for pain relief after inoculation. L-DOPA and benserazide were weighed and suspended in 0.3% carboxymethylcellulose (CMC-Na). The suspension (containing 200 mg/kg of L-DOPA and 75 mg/kg of benserazide) was orally administered by using an oral sonde once per day for 28 days. After the 28-day treatment period, the mice were deeply anesthetized with 1∼4% isoflurane and killed. The brain was perfused with PBS, fixed with 10% formalin neutral buffer solution for 7 days, and stored in 20% sucrose.

### Quantitation of α-Synuclein Pathologies in Mouse Brain

Eight brain slices for substantia nigra (bregma −2.8 ∼−3.8), eight slices for striatum (bregma +1.0 ∼ 0) and four slices for amygdala (bregma +1.0 ∼+1.5) at intervals of 100 mm from each of 10 independent mice were evaluated in order to cover the whole area of these brain regions. The sections were placed on coated slide glasses (PLATINUM PRO, Matsunami Glass Ltd.), dried, autoclaved at 105°C for 10 min, treated with 100% formic acid for 10 min, and stained with an anti-phosphorylated α-synuclein rabbit monoclonal antibody to phospho-Ser129 (PS129, Abcam, No. ab51253) and an rabbit antibody to tyrosine hydroxylase (TH) (Millipore, No. AB152). All experimental protocols were approved by the Animal Care and Use Committees of Tokyo Metropolitan Institute of Medical Science and Charles River Laboratories Japan, Inc., Statistical analysis was performed by using the unpaired Student’s *t*-test.

## Results

**FIGURE 1:**
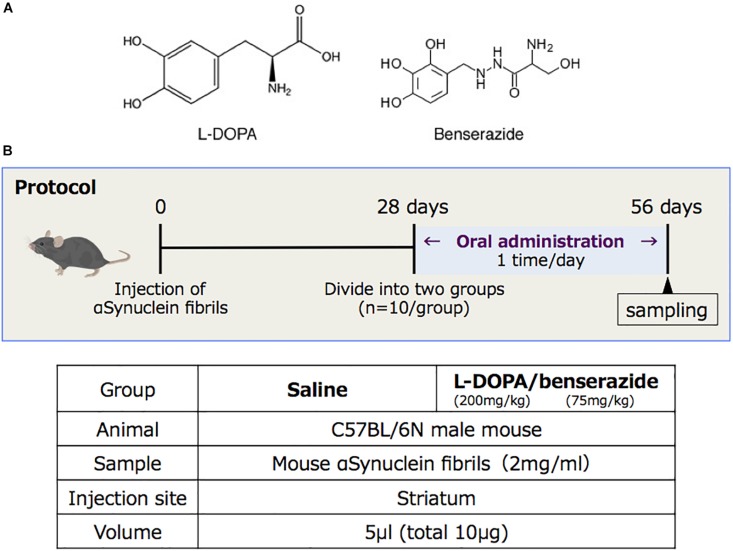
**(A)** Structures of L-DOPA and benserazide. **(B)** Schematic diagram of the experimental protocol of this study.

**FIGURE 2:**
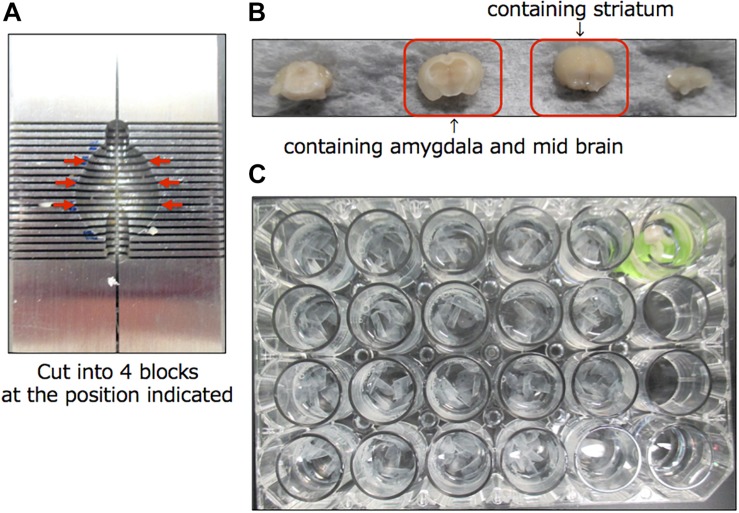
Preparation of brain sections for this study. **(A)** Mouse brain matrices (Bio Research Center Co., Ltd.) used in this study. Arrows indicate the positions where the cuts were made. **(B)** The cut blocks. **(C)** Serial frozen sections of these blocks (20 μm thickness) were prepared using a microtome and pooled in a 24-well dish.

**FIGURE 3:**
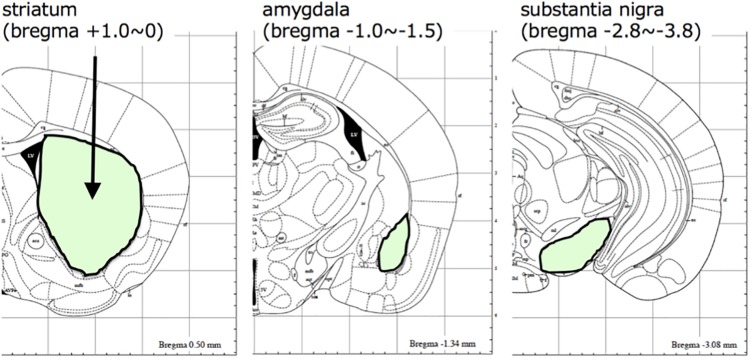
The brain regions evaluated in this study. Brain areas (striatum, amygdala, and substantia nigra) analyzed in this study are shown with their positions in the mouse brain atlas.

A schematic diagram of the experimental protocol of this study is shown in Figure 1. At 28 days after injection, the mice were randomly divided to two groups (10 mice each), which were given either saline or 200 mg/kg of L-DOPA and 75 mg/kg of benserazide (Figure 1A). Saline or L-DOPA/ benserazide was orally administered once per day for 28 days. After the 28-day treatment period, the mice were killed, and phospho-α-synuclein pathologies in the brain were examined (Figure 1B). We made this experimental schedule to evaluate the effect on α-synuclein propagation *in vivo* in a short period, because the injected fibrils degrade by 1∼2 weeks and the pathological α-synuclein starts to accumulate at 3∼4 weeks after injection in the condition. To quantitative pathological α-synuclein in these mouse brains, we carefully prepared the brain sections as shown in Figure 2. Fixed brains were coronally cut into four blocks using mouse brain matrices (Bio Research Center Co., Ltd.), and serial frozen sections of these blocks (20 μm thickness) were prepared using a microtome (Figure 3). To cover the whole area of these brain regions, eight sections at intervals of 100 μm were investigated for evaluation of striatum (bregma +1.0 ∼ 0) and substantia nigra (bregma −2.8 ∼−3.8), and four sections at intervals of 100 μm were used for evaluation of amygdala (bregma +1.0 ∼+1.5). The sections were pretreated with autoclaving and 100% formic acid, and stained with anti-phosphorylated α-synuclein rabbit monoclonal antibody to phospho-Ser129 (PS129). After staining, the areas of PS129-positive cells (μm^2^) in the ipsilateral sides of striatum, amygdala and substantia nigra were quantitated with a BZ-X Analyzer (BZ-H3C Hybrid Cell Count Software; Keyence), using a Keyence BZ-X710 fluorescence microscope (Keyence) at x10 magnification, with the same settings.

**FIGURE 4:**
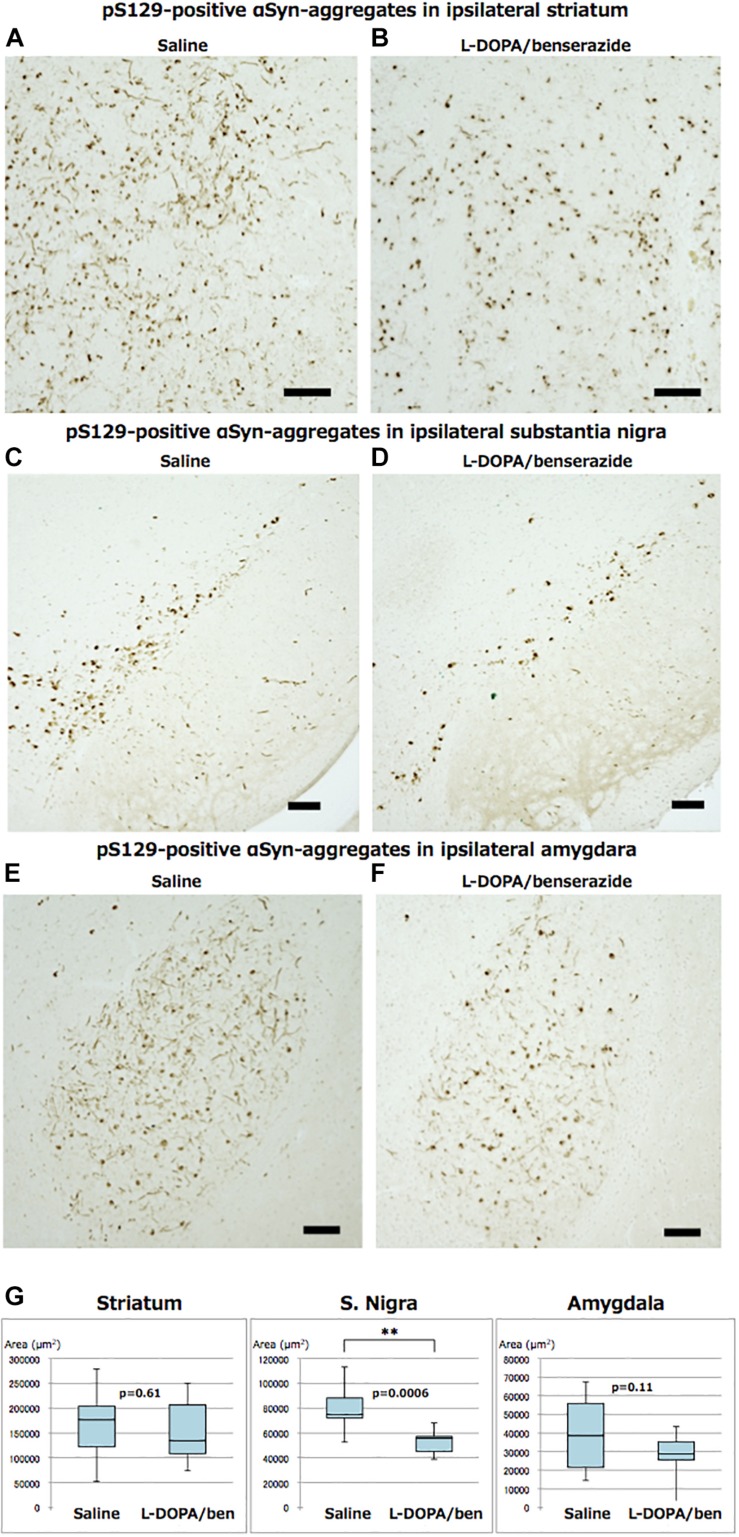
Immunostainings of ipsilateral sides of striatum, substantia nigra, and amygdala of mice with or without L-DOPA/benserazide treatment (**A–F**, bar 100 μm) and quantitation of PS129-positive aggregates **(G)**. The results of quantitation of PS129-positive aggregates are shown in box-plots. Data are presented as the median (horizontal bar) ± 25 and 75% quartiles (box) with the maximum and minimum (whiskers). Data were analyzed by Student’s *t*-test. *N* = 10 mice per treatment group. ^∗∗^*p* < 0.001.

Abundant phospho-α-synuclein pathologies were observed in striatum, substantia nigra, amygdala, cortex and various other brain regions, which are brain nuclei or regions providing direct input to striatum of mice injected with recombinant mouse α-synuclein fibrils, even at 2 months after injection, as expected (Figures 4A–F). The PS129-positive aggregates in the injected hemisphere of striatum, substantia nigra and amygdala were quantified and compared (Figure 4G). In striatum, mice treated with L-DOPA/benserazide showed no significant reduction in the amounts of phospho-α-synuclein pathologies, compared with the controls. In substantia nigra, however, the amounts of phospho-α-synuclein pathologies were significantly reduced in mice treated with L-DOPA/benserazide (*p* < 0.001). A reduction was also observed in amygdala, although it did not reach statistical significance (*p* < 0.1).

**FIGURE 5:**
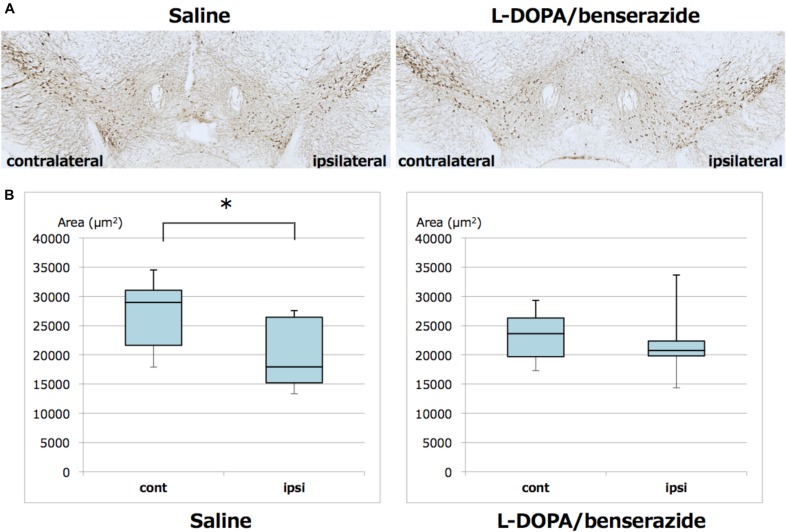
Quantitation of TH-positive neurons in the right and left hemispheres of substantia nigra of mice with or without L-DOPA/benserazide treatment. **(A)** Representative images of TH immunostaining of substantia nigra in mice injected with α-synuclein fibrils with or without L-DOPA/benserazide treatment. **(B)** Box-plots of TH-positive cells in these mice. Data are presented as the median (horizontal bar) ± 25 and 75% quartiles (box) with the maximum and minimum (whiskers). Data were analyzed by Student’s *t*-test. *N* = 8 mice per treatment group. ^*^p < 0.05.

We also quantitated TH-positive neurons in the ipsilateral and contralateral substantia nigra. TH immunochemical staining and analysis were performed in the same way as phosphorylated α-synuclein staining and analysis. In mice treated with saline, TH-positive neurons in the ipsilateral substantia nigra were significantly decreased compared to the contralateral region. However, this decrease of TH-positive neurons was suppressed in mice treated with L-DOPA/benserazide (Figure 5).

## Discussion

We and other groups previously showed that dopamine can inhibit aggregation of α-synuclein *in vitro* (Masuda et al., 2006; Ono et al., 2007, 2013), but it was not established whether this is also the case *in vivo*. Therefore, the present study was planned to investigate whether or not L-DOPA inhibits α-synuclein aggregation and/or propagation in brains of WT mice injected with preformed α-synuclein fibrils. Taken together, the results strongly suggest that L-DOPA/benserazide can suppress the aggregation of α-synuclein in substantia nigra and/or the propagation of pathological α-synuclein in brains of the mice, thus providing the first evidence that this treatment can ameliorate α-synuclein pathologies *in vivo*. Specifically, phospho-α-synuclein pathologies were significantly reduced in substantia nigra of mice treated with L-DOPA/benserazide. A similar tendency was also observed in amygdala, although not at the injection site (striatum), suggesting that the propagation of α-synuclein from the inoculation site to the connected regions is inhibited. These observations are consistent with the fact that administration of the drugs was started 1 month after inoculation of α-synuclein fibrils, when abnormal α-synuclein pathologies had already been generated in striatum and were propagating to substantia nigra, amygdala and cortex. We do not yet know whether the treatment works on normal α-synuclein or inhibits the transmission of abnormal α-synuclein. The nigrostriatal pathway is one of the major dopaminergic pathways, and is involved in movement. Indeed, loss of dopaminergic neurons and accumulation of phospho-α-synuclein in the substantia nigra region are major pathological features of PD. The finding that L-DOPA/benserazide significantly reduced pathologies at this location may indicate that L-DOPA or dopamine generated by decarboxylation of L-DOPA directly binds to abnormal or normal forms of α-synuclein and inhibits aggregation or indirectly inhibits the conversion of normal α-synuclein to an abnormal form in dopaminergic neurons. The excess amount of dopamine may protect TH neurons from α-synuclein aggregates by altering cell metabolism, such as protein synthesis, in TH-neurons. Curiously, we noticed that the overall staining of TH-neurons of mice treated with L-DOPA seemed to be weaker than that in saline-administered mice. In fact, the levels of TH-positive neurons in mice treated with L-DOPA/benserazide seemed to be slightly reduced compared to those in control mice. A plausible explanation for this is that the administration of L-DOPA reduces the requirement for TH to produce L-DOPA from L-tyrosine in the dopamine biosynthetic pathway, leading to an overall decrease in expression. Interestingly, a decrease of dopamine transporter in the striatum was observed in patients treated with 600 mg/day in ELLDOPA study (Fahn et al., 2004). In this study, 200 mg/kg/day L-DOPA was orally administered to mice. The human-equivalent dose is about 970 mg/day (for 60 kg human) by calculation based on body surface area (Nair and Jacob, 2016). The dose is ∼1.5 times higher than 600 mg/day but less than the maximum dose (1500 mg/day). It is also possible that L-DOPA/benserazide may have an effect on the expression or total level of endogenous α-synuclein in TH-neurons, leading to a reduced level of aggregation. Since anti-sense oligonucleotide (ASO) therapies are expected to be effective against various neurodegenerative proteinopathies by reducing the expression of targeted proteins, it is important to know whether or not there is any effect on the expression α-synuclein. Further studies will be needed to clarify the effective doses and mechanisms involved. We have not tested the efficacy of carbidopa, but it may have a similar effect to benserazide, since carbidopa is also often used with L-DOPA as an extracerebral decarboxylase inhibitor, and no significant difference in therapeutic effects or adverse reactions compared to benserazide has been reported. It will be interesting to see whether other drugs used in PD therapy, such as dopamine agonists or MAO-B inhibitors, also have similar effects on the propagation of α-synuclein in this model.

In this study, we focused on the effect of L-DOPA on the aggregation and propagation of pathological α-synuclein and did not perform any behavioral tests. One of the reasons for this was that it is difficult to detect motor dysfunctions of these WT mice even at 3 months after injection of α-synuclein fibrils. Thus, we cannot discuss the relationship between behavioral changes of L-DOPA-treated mice and propagation of α-synuclein. To investigate this issue, it would be helpful if biomarkers correlating with propagation could be identified. The development of sensitive and specific PET imaging probes for α-synuclein would also be useful to monitor the distribution of pathological α-synuclein. Although further studies are needed to examine the effects of L-DOPA in uninjected mice and other PD animal models, this α-synuclein transmission model using WT mice reproduces similar lesions without the need for a nucleation process, which is the rate-limiting step of these types of abnormal protein aggregations. Therefore, this model is considered to be suitable for evaluation of α-synuclein aggregation inhibitors, PET ligands, and ASO. Our findings here provide evidence that L-DOPA has an effect not only as a remedy for Parkinson’s disease palliation, but also as a disease-modifying drug by inhibiting α-synuclein aggregation and transmission, and further offer a compelling molecular rationale for the fact that administration of L-DOPA maintains motor functions for a long period, leading to improvement of QOL and prolongation of the survival time of PD patients. We believe that these findings and the mouse model used here will be helpful for the discovery and evaluation of new drugs to treat PD and related disorders.

## Conclusion

Oral administration of L-DOPA/benserazide suppressed the propagation of pathological α-synuclein to substantia nigra in mice injected with mouse α-synuclein fibrils into striatum. We present the first evidence that L-DOPA/benserazide ameliorates α-synuclein pathologies *in vivo*, although functional mechanisms of L-DOPA/benserazide treatment on α-synuclein pathologies are still unclear. The experimental protocols and detection methods used here are expected to be useful for evaluation of drug candidates or new therapies targeting the propagation of α-synuclein and other pathological proteins.

## Data Availability

All datasets generated for this study are included in the manuscript and/or the supplementary files.

## Ethics Statement

All experimental protocols were approved by the Animal Care and Use Committees of Tokyo Metropolitan Institute of Medical Science and Charles River Laboratories Japan, Inc.

## Author Contributions

AS designed the study, carried out the experiments, analyzed the data, and wrote a draft of the manuscript. YF, MT, HK, YT, and MS carried out the experiments and analyzed the data. S-iH critiqued the manuscript. MH designed the study, analyzed the data, and wrote the manuscript.

## Conflict of Interest Statement

YT and MS were employed by Charles River Laboratories Japan, Inc. The remaining authors declare that the research was conducted in the absence of any commercial or financial relationships that could be construed as a potential conflict of interest.
